# 

**Published:** 1889-06

**Authors:** 


					﻿Vol. IX.
JUNE, 1889.
NO. 6?
JFHE
JIOMCEOPATHIC PHYSICIAN,
A Monthly Journal of Homoeopathic Materia Medica
and Clinical Medicine.
“He is a freeman whom truth makes free, and all are slaves beside?’
“Seek the Truth: come whence it may, cost what it wiU,’\}''
EDITED AND PUBLISHED BY
Edmund j. lee, jme. e>.,
AND
WALTER M. JAMES, M. D.
PHILADELPHIA:
No. 1123 SPRUCE STREET.
X.O3STX» O KT
ALFRED HEATH & CO., SotE Agents foe Geeat Britain,
114 Ebury Street, Eaton Square.
Yearly Subscription, $2.50, invariably in advance. Single numbers, 25 cts.
Single numbers containing the Repertory Supplement, 50 Cts,
Entered at the Philadelphia Peet-Office as Second-Class Mail Matter.
				

